# Comparative Analysis Highlights Uniconazole’s Efficacy in Enhancing the Cold Stress Tolerance of Mung Beans by Targeting Photosynthetic Pathways

**DOI:** 10.3390/plants13141885

**Published:** 2024-07-09

**Authors:** Mingming Chen, Shuangfeng Dai, Daming Chen, Peiyi Zhu, Naijie Feng, Dianfeng Zheng

**Affiliations:** 1 College of Coastal Agricultural Sciences, Guangdong Ocean University, Zhanjiang 524088, China; sfdai_0426@163.com (S.D.); 15976563623@stu.gdou.edu.cn (D.C.); kaishineijuan@stu.gdou.edu.cn (P.Z.); 2 Shenzhen Research Institute of Guangdong Ocean University, Guangdong Ocean University, Shenzhen 518108, China

**Keywords:** soybean, mung bean, uniconazole, cold stress

## Abstract

Soybean (*Glycine max*) and mung bean (*Vigna radiata*) are key legumes with global importance, but their mechanisms for coping with cold stress—a major challenge in agriculture—have not been thoroughly investigated, especially in a comparative study. This research aimed to fill this gap by examining how these two major legumes respond differently to cold stress and exploring the role of uniconazole, a potential stress mitigator. Our comprehensive approach involved transcriptomic and metabolomic analyses, revealing distinct responses between soybean and mung bean under cold stress conditions. Notably, uniconazole was found to significantly enhance cold tolerance in mung bean by upregulating genes associated with photosynthesis, while its impact on soybean was either negligible or adverse. To further understand the molecular interactions, we utilized advanced machine learning algorithms for protein structure prediction, focusing on photosynthetic pathways. This enabled us to identify LOC106780309 as a direct binding target for uniconazole, confirmed through isothermal titration calorimetry. This research establishes a new comparative approach to explore how soybean and mung bean adapt to cold stress, offers key insights to improve the hardiness of legumes against environmental challenges, and contributes to sustainable agricultural practices and food security.

## 1. Introduction

In the realm of agricultural production and dietary staples, legumes are surpassed only by cereals in terms of their importance for human nutrition. They also play a pivotal role in global food systems [1,2,3]. This diverse family of plants encompasses a wide array of species, among which the soybean (*Glycine max*) and the mung bean (*Vigna radiata*) are particularly noteworthy. These species are not only integral to the agricultural economies of regions spanning from Asia to Africa but are also central to the diets within these cultures [4,5]. The nutritional profile of these legumes is highly esteemed, with soybeans containing as much as 25% protein by weight—a substantial contribution to the dietary protein intake [6]. Mung beans, on the other hand, provide approximately 22% protein and are often considered a lower-calorie alternative, making them a versatile option for diverse dietary needs and preferences [6,7]. The high protein content, coupled with the dietary fiber richness in these legumes, underscores their essential role in meeting the nutritional requirements of a significant portion of the global population, particularly for populations that rely on plant-based proteins as a dietary staple. Their nutritional importance, combined with their adaptability to a range of climates and soil conditions, cements the status of soybean and mung bean as agricultural cornerstones in both subsistence and commercial farming sectors. Their extensive cultivation is a testament to their adaptability and the crucial role they play in sustainable agricultural practices and food security initiatives worldwide.

Temperature, as a critical environmental parameter, exerts a profound influence on plant growth and development, often serving as a selective force that drives evolutionary adaptation and divergence among species [8,9]. Cold stress includes chilling (0–15 °C) and freezing (<0 °C), which cause varying degrees of harm to plants [10,11]. In the context of legume cultivation, both soybean and mung bean exhibit specific thermal preferences that dictate their geographic distribution and seasonal growth cycles [2,12,13,14]. The mung bean thrives under the balmy climes of warm seasons, displaying an acute susceptibility to suboptimal temperature conditions, particularly frost. The optimal temperature range for mung bean cultivation is a narrow window between 26 and 33 °C, beyond which the plants’ physiological processes and crop yields are compromised [15]. While the soybean showcases a broader thermal tolerance, capable of sustaining growth across a temperature spectrum from 24 to 36 °C, it is not impervious to the stresses induced by colder climates [16,17]. When exposed to temperatures falling below the 20 °C threshold, soybeans exhibit a marked decline in physiological functions such as imbibition rates—a crucial process for seed germination and water uptake—as well as embryonic development and overall metabolic activity, ultimately affecting respiration efficiency [18,19]. These stress responses at suboptimal temperatures reflect the underlying biochemical and cellular adaptations that these species have evolved, which may have far-reaching implications for their survival and productivity.

For the mung bean, enduring cold stress is particularly deleterious and has the potential to cause irreversible cellular damage and drastic reductions in yield, thereby imposing a significant constraint on its cultivation in cooler regions. In contrast, soybeans, while more cold-tolerant than mung beans, also suffer adverse effects when faced with low temperatures [20,21]. Cold stress in soybeans can lead to delayed germination, stunted seedling growth, and a decrease in nitrogen fixation, which is crucial for plant development and yield [22]. Furthermore, cold temperatures have been found to alter the expression of critical genes in soybeans related to lipid metabolism, cell wall modification, and hormonal signaling, suggesting an intricate network of stress responses [23,24,25]. Like mung beans, soybeans are susceptible to oxidative stress under cold conditions, which can cause cellular damage and compromise plant health [26,27,28]. This inherent vulnerability to cold stress highlights a critical aspect of the phenology of both mung bean and soybean, as well as their adaptability to changing climatic conditions. However, despite the recognition of temperature as a fundamental growth determinant for these legumes, research into the comparative physiological and molecular responses of soybean and mung bean to cold stress is lacking. Addressing this knowledge gap is paramount for enhancing our understanding of their adaptive mechanisms and for the development of strategies to mitigate the impacts of cold stress on these vital crops. Insights into genetic markers associated with cold tolerance in legumes offer promising directions for breeding more resilient cultivars [29,30]. Such research efforts are essential for informing selective breeding programs and for refining agricultural practices to bolster crop resilience amidst global climate change.

The susceptibility of legumes to low temperatures necessitates the development of strategies to enhance their resilience to cold stress, a critical concern for maintaining crop yields in the face of fluctuating climates. Within the arsenal of agricultural interventions, chemical regulators have emerged as a prominent tool for ameliorating the adverse effects of cold exposure in plant species. Notably, compounds like abscisic acid (ABA) are well-documented for their role in inducing cold tolerance, helping plants to withstand the physiological strains imposed by lower temperatures [31,32,33], Similarly, brassinosteroids (BRs) have been identified as potent enhancers of photosynthetic efficiency and antioxidant defense mechanisms, particularly in tomato plants subjected to chilling temperatures [34]. Salicylic acid (SA) has also been recognized for its protective effects under cold stress, primarily through its capacity to minimize cellular damage and curtail the accumulation of reactive oxygen species [35]. Parallel to these findings, methyl jasmonate (MeJA) has been shown to elevate cold tolerance by augmenting the levels of soluble sugars and proteins with antioxidant properties [36,37] as demonstrated by its role in preserving the quality of bananas during cold storage [38]. In a similar vein, uniconazole, a lesser-known plant growth regulator, has shown promise in mitigating the effects of cold stress in coix seedlings, pointing to its potential utility in broader applications [39]. Exogenous uniconazole pretreatment has been proven to enhance the cold tolerance of mung beans by improving the antioxidant enzyme activities, increasing non-enzymatic antioxidant content, and reducing the decrease in mung bean yield [40]. Similarly, spraying uniconazole on soybeans at the flowering stage could promote its tolerance to cold stress [41]. However, although the important role of uniconazole in plant resistance to cold stress has been revealed in a few species, and some physiological mechanisms have been elucidated, its molecular mechanisms have not yet been uncovered.

Soybeans and mung beans are important leguminous plants that are suitable for growing in warm environments, which limits their distribution range [26,42]. Moreover, leguminous plants are particularly sensitive to low temperatures during flowering, and encountering low temperatures during flowering can greatly reduce yield [40,43]. Therefore, in order to dissect the cold stress response mechanisms of soybean and mung bean, with a particular focus on the application of uniconazole as a stress alleviation agent, we designed four experiment groups: (1) Control Group (CK), sprayed with distilled water and kept at normal temperature; (2) Uniconazole Treatment Group (CK + U), sprayed with uniconazole and kept at normal temperature; (3) Cold Stress Group (LT), sprayed with distilled water and subjected to low temperature; and (4) Uniconazole + Cold Stress Group (LT + U), sprayed with uniconazole and subjected to low temperature. Transcriptome sequencing and widely targeted metabolomics were performed on materials from these groups. By conducting a detailed comparative analysis, this research sought to illuminate the multifaceted strategies employed by these legumes to navigate the challenges posed by low-temperature conditions, laying a solid foundation for cultivating cold resistant legume varieties and expanding the planting range of legume plants.

## 2. Results

### 2.1. Transcriptional and Metabolic Alterations under Cold Stress

**Figure 1:**
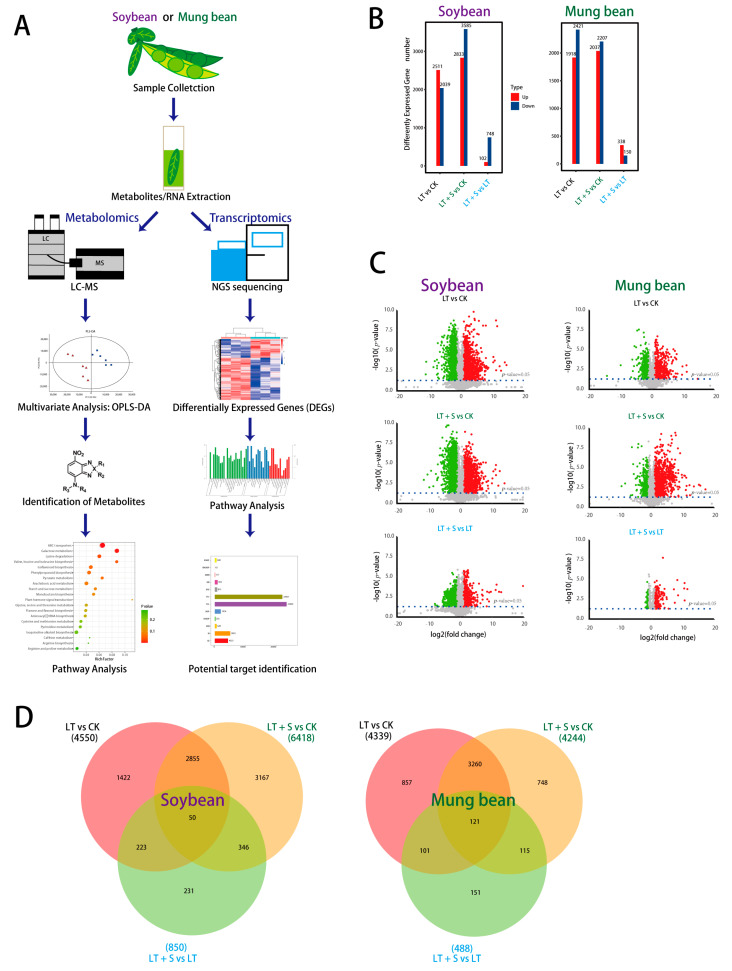
Workflow overview and comparative analysis of metabolome and transcriptome. (**A**) Transcriptomic and metabolic analysis schematic. (**B**) Quantitative overview of differentially expressed genes in soybean and mung bean. (**C**) Volcano plot highlighting up-regulated (in red) and down-regulated (in green) metabolites. (**D**) Venn diagram illustrating the overlap and differences in gene expression between various treatments in soybean and mung bean. CK represents the control group sprayed with water and planted under normal temperature, LT represents the group sprayed with distilled water and planted under low temperature, LT + S represents the group sprayed with uniconazole S3307 and planted under low temperature.

In exploring the systemic response to cold stress, RNA sequencing and liquid chromatography-mass spectrometry (LC-MS) analyses were performed to capture the transcriptional and metabolic shifts experienced by soybean and mung bean (Figure 1A). This was complemented by investigating the role of uniconazole S3307 as a potential mitigator of cold stress, which notably improved the retention of starch (Figure S1A) and sucrose (Figure S1B) in mung bean, as indicated by preliminary experiments.

For our experimental design, we chose 15 °C as the cold stress temperature based on the optimal growth range of mung bean and soybean, which begins at 25 °C. This 10 °C reduction is substantial enough to simulate a cold stress environment while remaining within a realistic range these crops might experience. This methodology is supported by research indicating marked physiological changes in these legumes under similar temperature deviations [16,17]. In addition, day 4 post-stress imposition was pinpointed as a critical time point, exhibiting the most pronounced effects of cold exposure (Figure S1A,B). At this juncture, a comparative RNA sequencing highlighted significant downregulation of 2039 genes in soybean and 2421 genes in mung bean (Figure 1B). Notably, the application of uniconazole S3307 led to an augmented downregulation of genes in soybean, while in mung bean, there was a slight reduction in the number of downregulated genes. This suggests a species-specific modulatory effect of uniconazole under cold stress (Figure 1B). A cross-comparison of the transcriptional landscape between cold stress alone and in conjunction with uniconazole treatment revealed a higher number of upregulated genes in soybean than in mung bean, further suggesting differential regulatory effects exerted by uniconazole (Figure 1D).

Metabolomic analysis mirrored these transcriptomic changes, with a less pronounced alteration in the metabolite profiles of mung bean compared to soybean, aligning with the more significant transcriptomic perturbations observed in soybean (Figure 1C). These findings collectively indicate that cold stress elicits distinct transcriptional and metabolic responses in soybean and mung bean, with uniconazole S3307 exerting differential effects on each species.

### 2.2. Uniconazole’s Impact on Photosynthesis under Cold Stress

**Figure 2:**
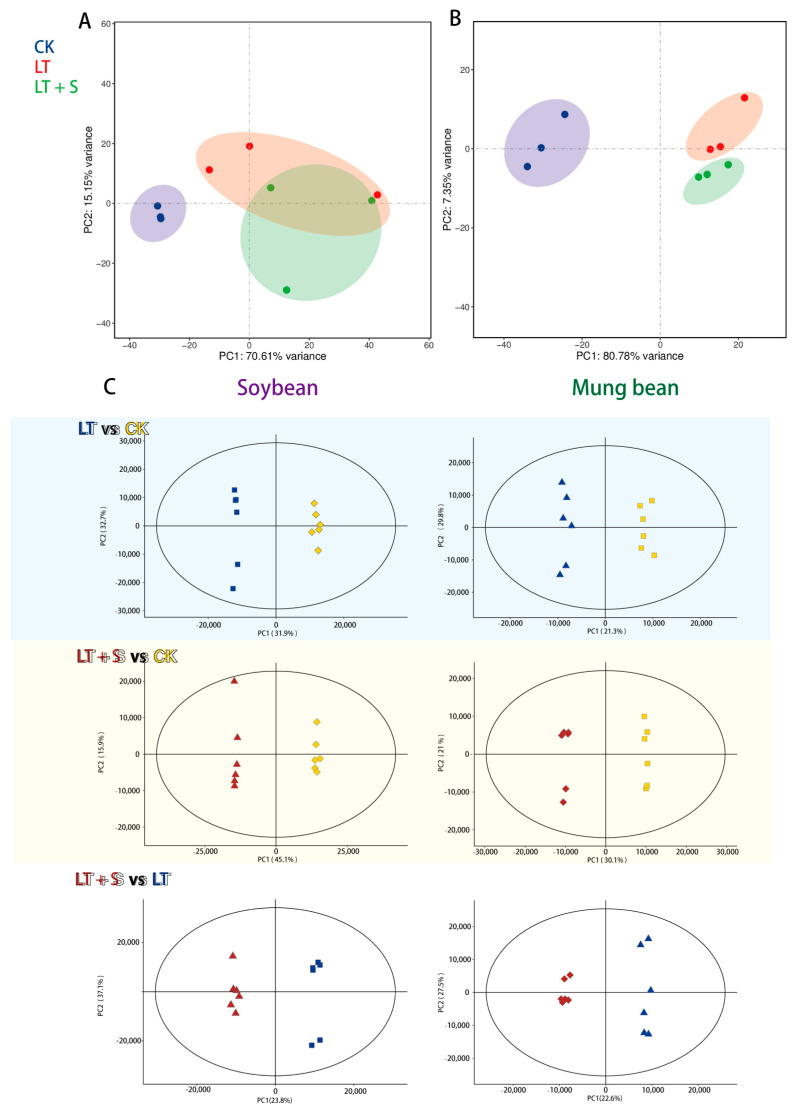
Principal component analysis (PCA) of soybean and mung bean response to various treatments. (**A**) PCA analysis of soybean transcriptomic profiles. (**B**) PCA analysis of mung bean transcriptomic profiles. (**C**) PCA analysis of metabolome expression profiles in soybeans and mung beans. CK represents a control group treated neither with chemical treatment nor cold stress, LT represents the group treated with cold stress without uniconazole S3307, and LT + S represents the group treated with uniconazole S3307 followed by cold stress.

Principal component analysis (PCA) was employed to decipher the broader implications of these transcriptional and metabolic changes. Both species exhibited clear segregation between the low-temperature treatment and control groups in both transcriptomic (Figure 2A,B) and metabolomic analyses (Figure 2C), underscoring the substantial impact of cold stress. Intriguingly, only in mung bean did uniconazole S3307 treatment result in a discernible shift away from the low-temperature stress profile at the transcriptomic level (Figure 2B).

To understand the underlying biochemical pathways driving these observations, KEGG pathway enrichment analysis was performed. Distinct pathways were differentially regulated in soybean and mung bean in response to cold stress and uniconazole treatment. In soybean, the citrate cycle (TCA cycle) was notably enriched under cold stress, whereas this was not observed in mung bean. Furthermore, flavone and flavanol biosynthesis pathways were enriched in mung bean under cold conditions, a response absent in soybean. The addition of uniconazole S3307 also differentially impacted purine metabolism in soybean and galactose metabolism in mung bean, suggesting that the compound may exert species-specific effects by modulating distinct metabolic pathways (Figure S2).

**Figure 3:**
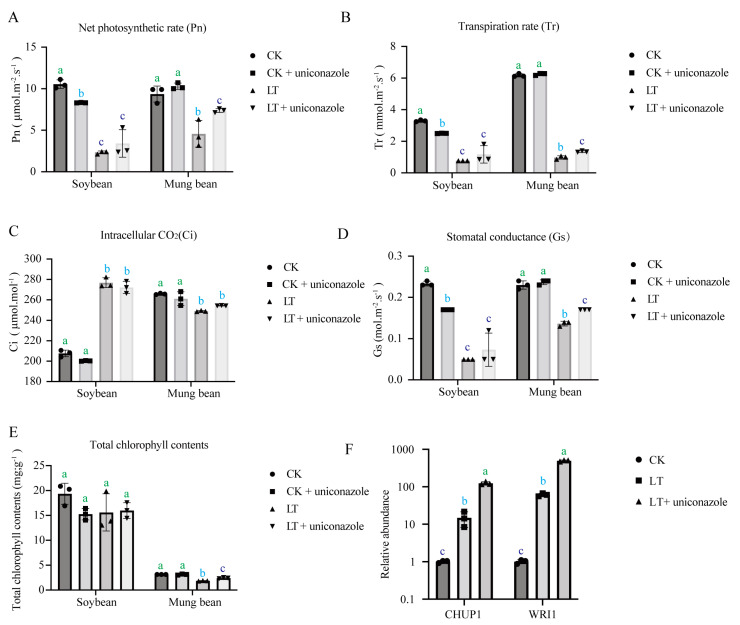
Impact of uniconazole on photosynthesis. Physiological analysis under different treatments: (**A**) net photosynthetic rate (Pn), (**B**) transpiration rate (Tr), (**C**) intracellular CO_2_ (Ci), (**D**) stomatal conductance, (**E**) total chlorophyll content, (**F**) RT-qPCR expression levels of CHUP1 and WRI1 Genes. CK represents a control group treated neither with chemical treatment nor cold stress, CK + uniconazole represents a group treated with uniconazole S3307 under normal temperature, LT represents a group treated with cold stress without uniconazole S3307, and LT + uniconazole represents a group treated with uniconazole S3307 followed by cold stress. Different letters on the bar chart indicate significant differences among treatments (*p* < 0.05, Duncan’s multiple range test).

Delving deeper into the physiological response, we focused on genes within the photosynthesis pathway that were among the top 20 regulated genes under uniconazole treatment. KEGG enrichment analysis revealed that uniconazole upregulated photosynthetic pathways in mung bean but downregulated them in soybean (Figure S3A,B). This aligns with physiological assessments where mung bean displayed significant recovery in photosynthetic parameters upon uniconazole application, whereas soybean did not exhibit such an improvement (Figure 3A). Several other physiological indicators including transpiration rate (Tr) (Figure 3B), stomatal conductance (Gs) (Figure 3D), and total chlorophyll content (Figure 3E) furtherly validated that uniconazole presented universal alleviating effects on mung bean under cold stress. Though no significant intracellular CO_2_ (Ci) (Figure 3C) alteration was detected under uniconazole treatment for both mung bean and soybean, it might suggest other sources of CO_2_, such as respiration function, compromised the limited change from photosynthesis. Quantitative PCR analyses confirmed that uniconazole significantly increased the transcription levels of photosynthesis-related genes CHUP1 and WRI1 in mung bean, reinforcing the potential of uniconazole as a facilitator of cold stress resilience through the modulation of photosynthesis (Figure 3B).

### 2.3. Unraveling the Differential Impact on Photosynthesis-Related Pathways in Soybean and Mung Bean under Uniconazole Treatment

**Figure 4:**
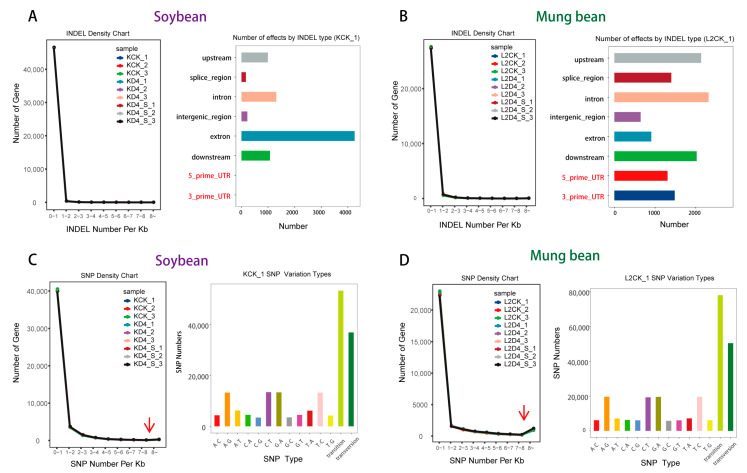
Comparative analysis of genetic variations and phylogeny in Asian beans. (**A**) INDEL events in soybean, (**B**) INDEL events in mung bean, (**C**) SNP occurrences in soybean, and (**D**) SNP occurrences in mung bean. The red arrows in Figure 4C,D point to the location where there is a significant difference in SNP density between soybean and mung bean.

Above all, the integrated comparative physiological and transcriptomic-metabolomic analysis offers a novel perspective on the distinct responses of soybean and mung bean to cold stress and uniconazole treatment, with a particular focus on photosynthesis-related pathways. To uncover the potential cause of this distinctive response, we initially explored genetic variations such as INDELs, SNPs, and alternative splicing (Figure S4) in both species. Though immediate variations due to cold stress or uniconazole were not evident, our analysis revealed a pronounced disparity in INDELs within the 5′ UTR and 3′ UTR regions between soybean and mung bean (Figure 4A,B), hinting at underlying genetic factors that might influence their differential response.

In soybean, the scarcity of INDELs contrasted with a higher occurrence in mung bean, potentially affecting gene expression related to photosynthesis under stress conditions. Additionally, while SNP enrichment was comparably distributed, mung bean showed a slightly elevated SNP frequency in critical genomic segments (Figure 4C,D), which could further contribute to its distinct response.

**Figure 5:**
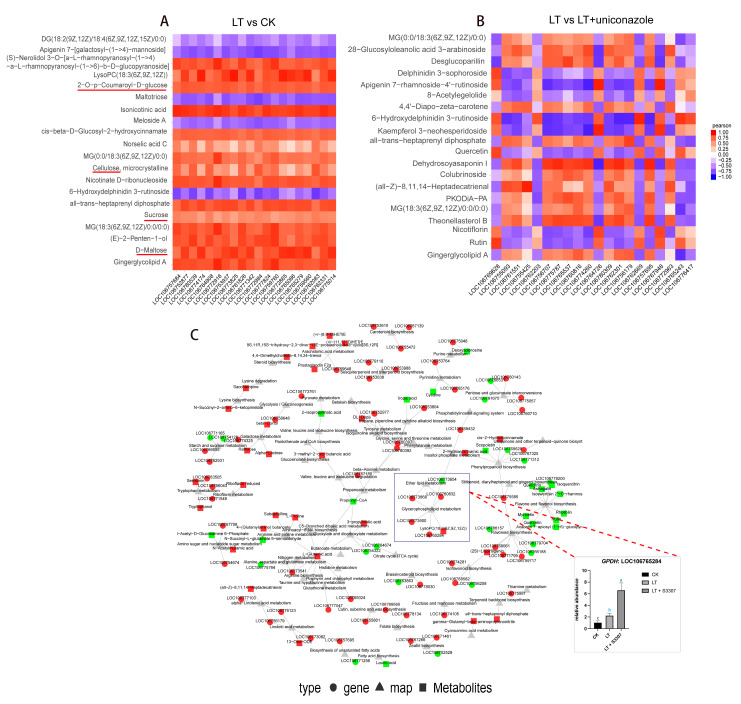
Interactive transcriptomic and metabolomic analysis in mung bean. (**A**) and (**B**) Pearson correlation test of top 20 metabolites and transcripts under different treatments. The sugar-related metabolites are underlined with red color in panel A. (**C**) KGML network analysis under low-temperature stress and uniconazole treatment with RT-qPCR validation of GPDH expression. CK represents a control group treated neither with chemical treatment nor cold stress, LT represents a group treated with cold stress without uniconazole S3307, and LT + uniconazole represents a group treated with uniconazole S3307 followed by cold stress. Different letters on the bar chart indicate significant differences among treatments (*p* < 0.05, Duncan’s multiple range test).

To probe deeper, we embarked on an integrative transcriptomic–metabolomic analysis in mung bean, particularly examining the interplay between photosynthesis and its critically related sugar metabolism under cold stress. This analysis revealed a coordinated alteration in sugar-related metabolic pathways, including those involving key sugars like glucose, maltose, and sucrose (Figure 5A, Supplementary Table S1). Significantly, upon the application of uniconazole, these pathways demonstrated a disrupted correlation, suggesting a notable restorative effect of uniconazole on photosynthesis-related pathways adversely affected by low temperatures (Figure 5B, Supplementary Table S2).

The KGML net plot analysis further reinforced this observation, showing uniconazole’s ability to counteract the global downregulation of genes and metabolites induced by cold stress (Figure 5C and Figure S5). These findings collectively underscore the complex genetic and metabolic interplay that governs the response of these species to environmental stressors, highlighting the pivotal role of photosynthesis-related pathways in their adaptation mechanisms.

### 2.4. Investigating Uniconazole’s Interaction with Photosynthesis-Related Proteins

Due to significant impact imposed by uniconazole on photosynthesis pathways, it was assumed that there should be a potential target involved in uniconazole targeting. To investigate the pronounced impact of uniconazole on the photosynthetic pathways of mung bean, our research focused on identifying the most susceptible targets of this chemical. We prioritized transcripts that showed significant changes in the presence of uniconazole, particularly those involved in photosynthesis, to determine whether there could be a direct binding effect. Due to a scarcity of published protein structures for these transcripts, we turned to a machine learning-powered approach, utilizing the AlphaFold algorithm for protein structure prediction, followed by molecular docking using AutoDock Vina. This innovative scheme of techniques allowed us to predict not just the structure of these proteins, but also their potential interactions with uniconazole.

**Figure 6:**
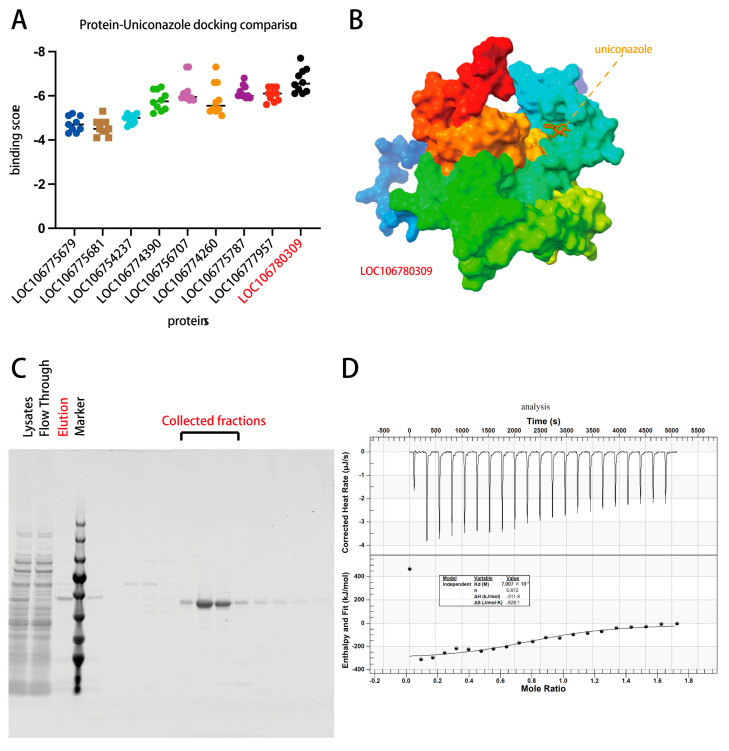
Assessment of uniconazole binding to photosynthesis-related genes. (**A**) Docking scores for uniconazole across photosynthesis-enriched gene sites. (**B**) Predicted binding site of uniconazole on LOC106777957. (**C**) Protein purification of LOC106777957. (**D**) Isothermal titration calorimetry (ITC) analysis of recombinant LOC106777957 interaction with uniconazole.

The results were quite revealing. It was discovered that uniconazole exhibited a strong affinity for certain photosynthesis-related proteins, as evidenced by high binding scores from our docking studies (Figure 6A). These findings suggest a targeted interaction, where uniconazole may be binding to proteins that are central to the photosynthetic process. Such interactions are graphically simulated and depicted in Figure 6B, indicating the sites where uniconazole might bind. Among the proteins we examined, the one corresponding to gene LOC106780309 stood out due to its remarkable binding affinity with uniconazole (Figure 6A). This gene encodes the chlorophyll a-b binding protein 13, a key component of the light-harvesting complex in mung bean. It plays a critical role in binding chlorophyll molecules, which is essential for capturing light and kick-starting the photosynthetic process that eventually leads to sugar production in the plant.

Given the absence of commercially available antibodies for the chlorophyll a-b binding protein 13, to confirm the binding with uniconazole, we resorted to purifying the recombinant protein (Figure 6C). This step was essential for performing isothermal titration calorimetry (ITC), a sensitive technique that measures the heat change thus reflecting the direct binding of two molecules, in this case, uniconazole and recombinant chlorophyll a-b binding protein 13. The ITC assay provided clear evidence that uniconazole specifically interacts with the chlorophyll a-b binding protein 13 showing a *K_d_* of 700 nM (Figure 6D), as opposed to the control protein (encoded by LOC106775679) (ITC not measurable), reinforcing the notion that uniconazole’s regulatory effect on the photosynthesis pathway is a significant mechanism by which it imparts cold stress tolerance in mung bean. However, while our findings shed light on the direct interaction between uniconazole and chlorophyll a-b binding protein 13, further studies are necessary to unravel the precise nature of this interaction under cold stress conditions. Understanding this could pave the way for new approaches to enhance legumes resilience to environmental stresses.

## 3. Discussion

Cold stress encompasses chilling stress (0–15 °C) and freezing stress (<0 °C), both of which pose significant threats to the growth, development, and yield of plants [11,44]. Different plants adapt to different growth temperature. Soybean and mung bean are valuable warm-season grain legume crops and are sensitive to low temperatures [41,45]. The damages caused by chilling stress observed in plants mainly include reduction in electron transport and photosynthetic efficiency [41,46] coupled with modifications in protein structure and enzyme activities [40]. In our research, we observed a decrease in chlorophyll content and stomatal conductance (Gs) in soybeans (Figure 3), which was consistent with previous research [41]. Despite the rise in intercellular carbon dioxide concentration observed in soybeans during cold stress, the overall net photosynthetic rate decreased, suggesting a decreased carbon dioxide utilization efficiency by soybeans under cold stress. In mung beans, we found similar trends in net photosynthetic rate (Pn), stomatal conductance (Gs), and transpiration rate (Tr) under cold stress, except for the intercellular carbon dioxide concentration, which implied that soybean and mung bean may face the cold stress in different ways. Furthermore, we observed that when plants treated with uniconazole were grown under normal temperatures, the net photosynthesis of soybeans decreased, while that of mung beans increased. However, when exposed to low temperatures after uniconazole treatment, both soybeans and mung beans exhibited higher net photosynthetic rates compared to the control group sprayed with water (Figure 3A). This suggests that the mechanism by which uniconazole enhances photosynthesis in soybeans was only triggered under cold stress. In contrast, mung beans exhibit an enhancement in photosynthesis regardless of cold stress conditions, with a more pronounced effect observed under cold stress.

The detrimental effects of cold stress on legume crops underscore the urgency to understand and improve plant tolerance mechanisms [47,48]. Legumes are pivotal for global nutrition and ecological sustainability, yet their productivity is compromised in the face of chilling temperatures. Current research has not fully unraveled the complex responses of these plants to cold stress, a gap attributed to the diverse cultivation environments and the distinct thermal adaptation strategies they exhibit [1,49]. This study’s comparative analysis of soybean and mung bean responses aims to illuminate these complexities. By integrating molecular, biochemical, and physiological data, it becomes evident that each legume species possesses a unique cold stress response signature. The findings particularly highlight how the application of uniconazole, a plant growth regulator, differentially influences their stress pathways, potentially offering a new avenue for enhancing cold tolerance in mung bean. It is posited that understanding these differential responses is crucial for designing targeted interventions, which could include selective breeding programs or agronomic practices tailored to bolster the inherent resilience of these legumes. Such strategies are imperative for safeguarding crop yields and ensuring the nutritional security that legumes provide, particularly in regions where climate change threatens traditional agricultural practices.

The transcriptomic variations between mung bean and soybean under cold stress conditions reveal intriguing species-specific responses, pivotal for understanding plant adaptation mechanisms. Mung bean’s distinct transcriptomic profile upon uniconazole treatment suggests an innate flexibility in its stress response system, which may confer an evolutionary advantage in variable climates. The upregulation of photosynthesis-related pathways in mung bean indicates a potential strategy for sustaining growth and productivity during cold spells, which is conspicuously absent in soybean. This divergence highlights the nuanced complexity of plant stress biology, emphasizing the importance of a tailored approach to crop management and improvement strategies. The differential efficacy of uniconazole, a known gibberellic acid synthesis inhibitor, underscores the selective nature of stress mitigators and their potential interactions with plant-specific physiological pathways. While mung bean benefits from uniconazole’s role in enhancing photosynthetic efficiency under cold stress, soybean does not exhibit the same level of responsiveness, hinting at a deeper metabolic and genetic divergence between the two species [41]. The physiological data corroborating these molecular findings not only validate the bioinformatics predictions but also open a discourse on the adaptive significance of such stress responses. The apparent resilience of mung bean to cold stress when treated with uniconazole presents a case for its use in agricultural practices where cold stress is a limiting factor. Conversely, the lack of a significant response in soybean to the same treatment at temperatures as low as 15 °C raises questions about the underlying reasons for its contrasting behavior.

The integration of transcriptomic and metabolomic data in mung bean highlights a synergistic interaction under cold stress, reflected in a marked correlation between gene expression and metabolite profiles. The application of uniconazole, however, appears to modulate this relationship, suggesting an influence on the gene regulatory mechanisms that are responsive to low temperatures. KGML pathway analysis supports the hypothesis that uniconazole may mitigate the suppressive effects of cold stress on gene and metabolite expression by targeting specific genes within these pathways (Supplementary Table S3). Notably, uniconazole’s role in inhibiting gibberellic acid synthesis, known to affect germination and growth, may also extend to modulating the photosynthesis pathways under cold stress conditions [50,51]. Protein interaction predictions, facilitated by the AlphaFold algorithm and molecular docking simulations, indicate strong binding affinities between uniconazole and key photosynthetic proteins, particularly the chlorophyll a-b binding protein 13, encoded by LOC106780309. This protein, essential for the light-harvesting complex’s function, was biochemically validated to interact with uniconazole through isothermal titration calorimetry, pointing to a direct role in enhancing cold stress tolerance. These findings propose a model where uniconazole potentially targets critical components of the photosynthesis machinery, thereby alleviating the deleterious effects of cold stress. In contrast, soybean’s lack of a similar response might be attributed to the absence of compatible molecular targets for uniconazole, a disparity that necessitates further molecular investigation to elucidate the mechanistic basis of these differential responses.

This research underscores the differential response of mung bean and soybean to cold stress and uniconazole treatment, highlighting mung bean’s ability to maintain and enhance photosynthetic activity under stress conditions. However, it is also important to acknowledge the limitations of this study, particularly the relatively small sample size, only one variety used and the controlled experimental conditions. Further field trials with larger sample sizes, more varieties, and conducted in multiple locations are necessary to validate these preliminary findings and ensure their applicability to real-world agricultural settings.

**Figure 7:**
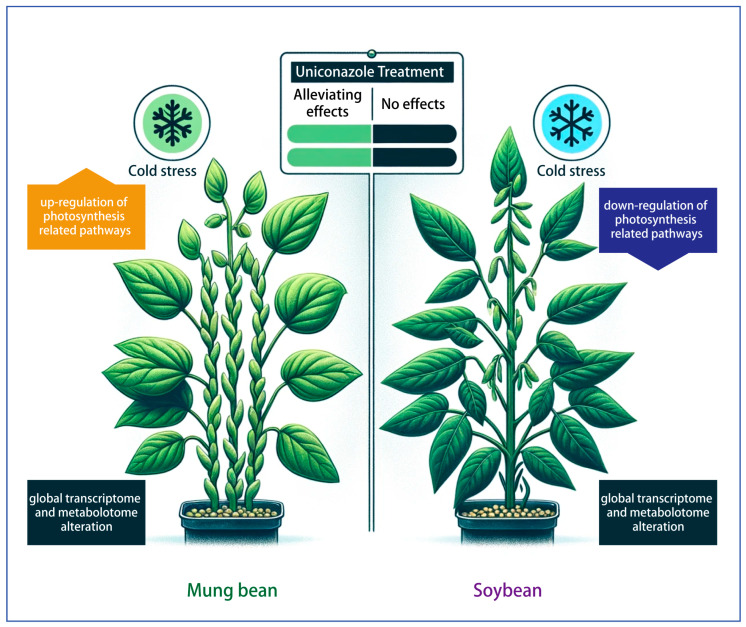
Comparative analysis of soybean and mung bean responses to uniconazole treatment under low-temperature stress conditions.

Overall, the study provides an enhanced understanding of the molecular dynamics underpinning the response of soybean and mung bean to low-temperature stress (Figure 7). Through the lens of advanced bioinformatics, this research has pinpointed pivotal photosynthesis-related genes potentially implicated in the resilience mechanisms activated by uniconazole. The insights gleaned from protein docking predictions pave the way for a deeper exploration of molecular interactions essential for cold stress adaptation. However, it is important to note that the conclusions drawn in this study were based on a single variety chosen from each species, and their genetic composition may not be universally applicable. Furthermore, the experimental setup took place in a greenhouse setting, which deviates from real field conditions. Hence, our research results need to be further validated in field experiments. While, as a foundation for future inquiries, this work proposes novel approaches to bolster legume tolerance to temperature extremes, a significant step forward in the pursuit of crop sustainability and security.

## 4. Materials and Methods

### 4.1. Plant Materials and Cultivated Condition

The mung bean variety ‘Lvfeng No.2′ and the soybean variety ‘Kenfeng No.16′ were procured from the Germplasm Bank of the National Coarse Grains Engineering Technology Research Center, China. Seeds, selected for uniformity in size and quality, were sown on 10 May 2022, placing three seeds equidistantly in each of five designated holes per pot. Post-germination, at the V2 developmental stage, plants were thinned to maintain one healthy seedling per hole. Standard agronomic practices were applied, including timely weeding and pesticide application to ensure optimal plant health. Plants were cultivated in 30 × 23 × 32 cm pots made from polyvinyl chloride resin (PVC), utilizing a soil mixture composed of cultivated land soil and sand in a 3:1 ratio. The meadow black soil employed for this study exhibited the following physicochemical properties: alkali-hydrolysable nitrogen at 158.8 mg·kg^−1^, available phosphorus at 30.8 mg·kg^−1^, available potassium at 163.7 mg·kg^−1^, and organic matter content of 27.6 g·kg^−1^, with a pH of 6.87.

### 4.2. Chemical and Stress Treatment

Plants were subjected to a chemical treatment involving uniconazole S3307 (95% purity) sourced from Jiangsu Qizhou Green Chemical Co., Ltd., Qizhou, China. At the onset of flowering, half of the plants (120 plants) were sprayed with 50 mg·L^−1^ uniconazole solution. The control group (120 plants) was treated with an equivalent volume of distilled water. Following a 36 h period post-treatment, 60 uniconazole-treated and 60 water-treated plants were transferred to a phytotron facility at the Heilongjiang Agricultural Academy for the low-temperature stress experiment (15/15 °C—day/night temperature, relative humidity (RH) of 75%, and a natural light photoperiod). Low-temperature treated plants were maintained at 15 °C for a continuous four-day period. The remaining 60 uniconazole-treated and 60 water-treated plants were placed under natural environments as controls. In conclusion, the plant materials of these two species were divided into 4 groups, denoted as CK (sprayed with water and normal temperature), CK + uniconazole (sprayed with uniconazole and normal temperature), LT (sprayed with water and low-temperature), LT + uniconazole (sprayed with uniconazole and low-temperature).

### 4.3. RNA-Seq and Transcriptomics Analysis

Leaf samples with different treatments (CK, LT, LT + S) were flash-frozen in liquid nitrogen immediately upon collection and subsequently stored at −80 °C. Each pot represented an independent biological replicate, with three replicates per treatment group to ensure robust statistical analysis. Total RNA was isolated from the harvested samples utilizing the Trizol reagent method, following the protocol previously described by Ingolia et al. [52]. The cDNA library was the constructed and then sequenced on the Illumina HiSeq Ten X platform. Quality control was conducted using Trimmomatic to remove poly-N sequences with default parameters [53]. The filtered reads then aligned to the respective reference genome by using hisat2 (v2.1.0) software [54], with soybeans aligned to the reference genome (http://ftp.ncbi.nlm.nih.gov/genomes/all/GCF/000/004/, 10 June 2021) and mung bean aligned to corresponding reference genome (https://ftp.ncbi.nlm.nih.gov/genomes/all/GCA/000/741/045/GCA_000741045.2_Vradiata_ver6/, 10 June 2021). Differential gene expression analysis utilized htseq-count for read quantification, with normalization by DESeq2 [55], targeting genes with a fold change greater than 2 and a *p*-value below 0.05. Identified genes were further analyzed for functional relevance using Gene Ontology (GO) and Kyoto Encyclopedia of Genes and Genomes (KEGG) pathway enrichment. GO and KEGG analysis were carried out by using the function enrichGO and enrichKEGG of R package clusterProfiler [56].

### 4.4. Sample Preparation and Metabolite Analysis

Fresh leaf samples from groups CK, LT, and LT + S, which were sampled from the same batch as transcriptome sequencing, were used for widely targeted metabolite assessments, ensuring the prompt and accurate measurement of response variables. Six biological replicates were performed for each treatment to avoid potential errors. The 80 mg accurately weighed samples were transferred to a 1.5 mL Eppendorf tube, which contained 1 mL of 70% methanol and 20 μL of 2-chloro-l-phenylalanine (0.3 mg/mL) dissolved in methanol as internal standard. Samples were placed at −20 °C for 2 min, then grinded at 60 HZ for 2 min, and ultrasonicated at ambient temperature for 30 min after vortexed, then placed at −20 °C overnight. Samples were centrifuged at 13,000 rpm, 4 °C for 10 min. The supernatants (150 μL) from each tube were collected using crystal syringes, filtered through 0.22 μm microfilters and transferred to LC vials. The vials were stored at −80 °C until LC-MS analysis.

An ACQUITY UHPLC system (Waters Corporation, Milford, CT, USA) coupled with an AB SCIEX Triple TOF 5600 System (AB SCIEX, Framingham, MA, USA) was used to analyze the metabolic profiling in both ESI positive and ESI negative ion modes. An ACQUITY UPLC BEH C18 column (1.8 μm, 2.1 × 100 mm) was employed in both positive and negative modes. The binary gradient elution system consisted of (A) water (containing 0.1% formic acid, *v*/*v*) and (B) acetonitrile (containing 0.1% formic acid, *v*/*v*) and separation was achieved using the following gradients: 0 min, 5% B; 0.5 min, 5% B; 5.5 min, 40% B; 8 min, 100% B; 11 min, 100% B; 11.5 min, 5% B; 15 min, 5% B. The flow rate was 0.4 mL/min and column temperature was 45 °C. All the samples were kept at 4 °C during the analysis. The injection volume was 2 μL.

Data acquisition was performed in full scan mode (*m*/*z* ranges from 100 to 1000) combined with IDA mode. Parameters of mass spectrometry were as follows: Ion source temperature, 550 °C (+) and 550 °C (−); ion spray voltage, 3000 V (+) and 2800 V (−); curtain gas of 35 PSI; declustering potential, 100 V (+) and −100 V (−); collision energy, 10 eV (+) and −10 eV (−); and interface heater temperature, 550 °C (+) and 600 °C (−). For IDA analysis, range of *m*/*z* was set as 25–1000, the collision energy was 30 eV.

The QCs were injected at regular intervals (every 10 samples) throughout the analytical run to provide a set of data from which repeatability can be assessed.

### 4.5. Assessment of Photosynthetic Gas Exchange Parameters

To elucidate the photosynthetic performance of soybean and mung bean under cold stress, key gas exchange parameters were measured. This was accomplished using the LI-6400 Portable Photosynthesis System (LI-COR Biosciences, Lincoln, NE, USA), a robust tool for evaluating plant physiological responses to environmental variables. The following parameters were assessed:

Net Photosynthetic Rate (Pn): This metric indicates the efficiency with which CO_2_ is converted to organic compounds during photosynthesis. Stomatal Conductance (Gs): Stomatal conductance serves as an indicator of the rate at which CO_2_ enters and water vapor exits the leaf, reflecting the plant’s water-use efficiency and gas exchange potential. Intercellular CO_2_ Concentration (Ci): The concentration of CO_2_ within the leaf intercellular space, providing insight into the CO_2_ availability for carboxylation. Transpiration Rate (Tr): The rate of water vapor release from the leaf, closely linked to stomatal behavior and plant water status.

The instrument settings were calibrated to simulate ambient light conditions with a photosynthetic photon flux density of 1200 μmol·m^−2^·s^−1^, closely mimicking full sunlight conditions. CO_2_ was supplied at a concentration of 400 μmol·mol^−1^ to match atmospheric levels, ensuring relevance to field conditions. Leaf temperature was maintained at 26 °C, optimal for soybean and mung bean photosynthesis, while relative humidity was controlled at approximately 25%, reflecting common growing conditions. These controlled measurements allow for the comparison of the intrinsic photosynthetic capacities of soybean and mung bean under standardized environmental conditions, thereby minimizing variability and enabling an accurate assessment of their response to cold stress.

### 4.6. Quantification of Total Chlorophyll Content

For the assessment of total chlorophyll content, fresh leaf samples from both soybean and mung bean plants were prepared. Precisely 0.2 g of mature leaves, deemed functionally active, were harvested, and finely sectioned into approximately 2 mm fragments to enhance solvent access to cellular contents. These fragments were immediately placed in test tubes containing 20 mL of 95% ethanol, which facilitates chlorophyll extraction. To prevent degradation and ensure the integrity of the chlorophyll, the samples were incubated for 24 h in a dark environment, preventing light-induced alterations.

Post-extraction, the samples underwent a comparative visual inspection to confirm the leaching of chlorophyll into the ethanol solvent. For quantification, a 2.5 mL aliquot of the ethanolic chlorophyll extract was carefully transferred into a quartz cuvette. Absorbance readings were taken at two distinct wavelengths, 665 nm and 649 nm, which correspond to the absorption maxima of chlorophyll a and chlorophyll b, respectively, using an ultraviolet-visible (UV-Vis) spectrophotometer. These readings enable the calculation of the total chlorophyll content (*CT*), utilizing the following equation:

*CT* = 6.63 × *A_665_* − 18.08 × *A_649_*

where *A_665_* and *A_649_* represent the absorbance at 665 nm and 649 nm, respectively. This spectrophotometric method provides a reliable measure of the chlorophyll concentration, which is indicative of the photosynthetic potential and the health status of the leaves under investigation.

### 4.7. Real-Time Quantitative PCR (RT-qPCR) Assay

To verify the accuracy of transcriptome sequencing, RT-qPCR was performed following RNA extraction as previously described [52]. Primer sequences were designed for target and reference genes utilizing Primer Premier 5.0 software to maximize the efficiency and specificity of amplification. The *β-Actin* gene was employed as an internal control to normalize expression data. The primers used are listed below:

*β-Actin* Forward (F): CGAACCACCAACCGTAGTAAA
*β-Actin* Reverse (R): CAGATGAGAATGCCCGAGAG
*CHUP1* Forward (F): GGCTGCTACCAAGATTTCTACC
*CHUP1* Reverse (R): AAGCGTCTCCTCCAGTGTCA
*WRI-1* Forward (F): TCACCACCCTCACCACATTC
*WRI-1* Reverse (R): TTCAGGGTTTGAGGAAGACATT
*GPDH* Forward (F): GGACCAATCACAGAATGACAAGT
*GPDH* Reverse (R): AAGTTAGAAGGACAGCACACACC

The reaction solution for RT-qPCR included 10 μL 2 × SG Fast qPCR Master Mix (High Rox, B639273, BBI, ABI), 0.5 μL of primer F (10 mM), 0.5 μL of primer R (10 mM), 1 μL of cDNA, and 8 μL of nuclease-free water, for a total of 20 μL volume. Amplification and detection were executed on the StepOne Plus Thermal Cycler (ABI, Foster, CA, USA). The relative gene expression levels were quantified employing the 2^−ΔΔCT^ method, which normalizes the target gene expression to the endogenous reference gene and provides fold change in expression relative to a control. Data acquisition and analysis were conducted with the thermal cycler’s integrated software, which includes quality control measures to verify amplification consistency and efficiency. The SPSS 23.0 was used to calculate the variance between groups and treatments, and the graph was made by R or Graphpad Prism 9 software. Three independent biological replicates and three technological replicates were conducted for each sample.

### 4.8. Detection of Novel Transcripts, Alternative Splicing, and Genetic Variations

Transcript quantification was executed using two complementary software packages: bowtie2 for aligning sequencing reads to the reference genome, and eXpress for estimating transcript abundance, expressed as fragments per kilobase of transcript per million mapped reads (FPKM) and raw read counts. Subsequent normalization of transcriptional data was performed using the “estimateSizeFactors” function within the DESeq [55] R package, which adjusts for varying library sizes between samples.

In the pursuit of novel transcript discovery, we utilized the hisat2 [57] algorithm to ensure high-efficiency read mapping. StringTie (v2.0) software then assembled RNA-sequencing reads into potential novel transcripts. The integrity and relevance of these assembled transcripts were verified by cuffcompare [58], which contrasts the novel transcriptome assembly against a known reference annotation. Criteria for novel transcripts included a minimum separation of 200 base pairs from known genes and a maximum transcript length of 180 base pairs to ensure specificity and to avoid annotation of spurious transcriptional artifacts.

For the comprehensive detection of alternative splicing events and genetic variations such as single nucleotide polymorphisms (SNPs) and insertions/deletions (INDELs), we employed a suite of specialized tools. ASprofile was utilized to analyze alternative splicing patterns, providing insights into the transcript diversity and potential regulatory mechanisms in response to cold stress. Samtools [59] and bcftools [60] were engaged to identify and quantify SNP and INDEL events, offering a genomic perspective of intraspecies variation and the potential impact of these genetic differences on cold stress response. This multifaceted approach enables a robust investigation into the genomic and transcriptomic adaptations of soybean and mung bean to cold conditions.

### 4.9. Protein Structure Prediction and Uniconazole Docking Analysis

To elucidate the molecular interactions between uniconazole and the proteins implicated in photosynthesis, we implemented a computational approach for structure estimation and ligand docking. Gene candidates showing significant upregulation in the KEGG pathway analysis related to photosynthesis were identified. The corresponding protein sequences were retrieved from the UniProt database, a comprehensive resource for protein sequence and annotation data.

Utilizing the state-of-the-art AlphaFold algorithm, we predicted the tertiary structures of the selected proteins [61,62]. This deep learning-based method has revolutionized the field of protein structure prediction, providing highly accurate models that are instrumental for understanding protein function and interactions. The three-dimensional structure of uniconazole was obtained in structure data file (SDF) format from the PubChem database (PubChem CID: 6436604), which serves as a public repository for molecular structures and bioactivity data. Subsequent molecular docking simulations were carried out using AutoDock Vina [63], a widely recognized tool for predicting the binding affinities and poses of small molecules to their protein target. The docking process was performed in a blind mode, allowing the exploration of potential binding sites across the entire surface of the protein structures.

### 4.10. Recombinant Protein Purification and ITC Affinity Test

*Escherichia coli* BL21 Star (DE3) cells, transformed with the pColdI-LOC106780309 (Vigan.07G123300.01) plasmid, were propagated in LB medium supplemented with ampicillin, induced with 1 mM IPTG at 15 °C, and stored at −80 °C. To extract the protein, cell pellets were resuspended in bacterial lysis buffer consisting of 20 mM HEPES-NaOH, 500 mM NaCl, 10 mM imidazole, 0.5% NP-40, and 10 mM β-mercaptoethanol, adjusted to pH 7.5. After sonication and centrifugation, the lysate was incubated with Ni-NTA agarose beads, washed with high-salt and low-salt buffers, and the His-tagged protein was eluted. Further purification via chromatography employed a gradient of buffer A (20 mM HEPES-NaOH, 10 mM NaCl, 10% glycerol, and 1 mM DTT) and buffer B (20 mM HEPES-NaOH, 1 M NaCl, 10% glycerol, and 1 mM DTT). Fractions containing the protein were buffer-exchanged and concentrated, and the final protein was stored in a storage buffer (20 mM HEPES-NaOH, 150 mM NaCl, 10% glycerol, and 1 mM DTT).

Isothermal titration calorimetry (ITC) was employed to analyze the binding interaction between recombinant proteins and the ligand, uniconazole. Both the protein and uniconazole were prepared in identical storage buffer conditions to negate the heat of dilution effects. The concentrations of the protein and ligand were determined accurately as 100 μM, with adjustments made to accommodate the expected binding affinity. The ITC experiment was conducted following the instrument’s calibration (MicroCal Malvern Panalytical, GE Healthcare Life Sciences), with the protein solution placed in the calorimeter cell and the ligand in the syringe. Automated titration was performed under optimized conditions including temperature (25 °C), injection volume (3 μL), and intervals (1 min). The resulting thermograms were analyzed using the instrument’s software, integrating the heat change per injection to derive the binding constants, stoichiometry, enthalpy, and entropy. Control experiments, including buffer injections, were conducted to validate the specificity and reproducibility of the binding interaction.

### 4.11. Statistical Analysis

The data about physiological indicator and gene expression were subjected to analysis of variance (ANOVA) and Duncan’s multiple range test at *p* < 0.05 significance level between different treatments with SPSS (21.0) software. The figures were drawn with R 4.2.3 software.

## 5. Conclusions

In this study, we examined the impact of uniconazole on varieties ‘Kenfeng No.16′ and ‘Lvfeng No.2′ under cold stress as an example to explore the mechanisms through which uniconazole bolsters soybean and mung bean resilience to cold stress. The results showed that uniconazole had significant efficacy in enhancing mung bean’s tolerance to cold stress by targeting and upregulating genes associated with photosynthetic pathways, while its impact on soybean was either negligible or adverse. Further research revealed that uniconazole exhibited a strong affinity for chlorophyll a-b binding protein 13, which will indirectly affect the synthesis of carbohydrates in mung beans. This may be the reason why uniconazole alleviates the harm of cold stress in mung bean. In summary, our study has preliminarily explored the molecular mechanisms of uniconazole in mung bean’s resistance to cold stress. However, due to the limited variety and sample size, as well as the lack of validation through multiple location experiments, future studies with larger sample sizes, more varieties, and multiple locations would be essential to validate and expand these preliminary findings.

## Data Availability

The datasets generated during and/or analyzed during the current study are available in the GEO (Gene Expression Omnibus) repository (GSE193328), and related scripts are available from the corresponding author on reasonable request.

## Supplementary Materials

The following supporting information can be downloaded at: https://www.mdpi.com/article/10.3390/plants13141885/s1; Figure S1: Uniconazole Improves Retention of Starch and Sucrose in Mung Bean Under Cold Stress; Figure S2: KEGG Pathway Enrichment Analysis of Soybean and Mung Bean Under Cold Stress and Uniconazole Treatment; Figure S3: Transcriptomic Changes in Photosynthesis Pathways in Soybean and Mung Bean Under Cold Stress and Uniconazole Treatment; Figure S4: Genetic Variation Analysis in Soybean and Mung Bean Under Cold Stress and Uniconazole Treatment; Figure S5: Integrative Transcriptomic-Metabolomic Analysis in Mung Bean Under Cold Stress and Uniconazole Treatment; Figure S6: Predicted Binding Sites of Uniconazole on Photosynthesis-Related Proteins in Mung Bean. Table S1. Correlation analysis of differentially expressed genes and metabolites in LT_vs_CK; Table S2: Correlation analysis of differentially expressed genes and metabolites in LT+S_vs_LT; Table S3: KEGG enrichment analysis on the differential expression genes between two groups (sprayed with uniconazole and water under cold stress) in mung beans.
