# Supplementary material for: Comparative Analysis Highlights Uniconazole’s Efficacy in Enhancing the Cold Stress Tolerance of Mung Beans by Targeting Photosynthetic Pathways
Source: Plants (Basel). 2024 Jul 9;13(14):1885. doi: 10.3390/plants13141885 (PMC11280120; doi:10.3390/plants13141885)
Supplement: Supplementary file 1 [file plants-13-01885-s001.zip › plants-3046194-supplementary-update/Figure-S6.pdf]

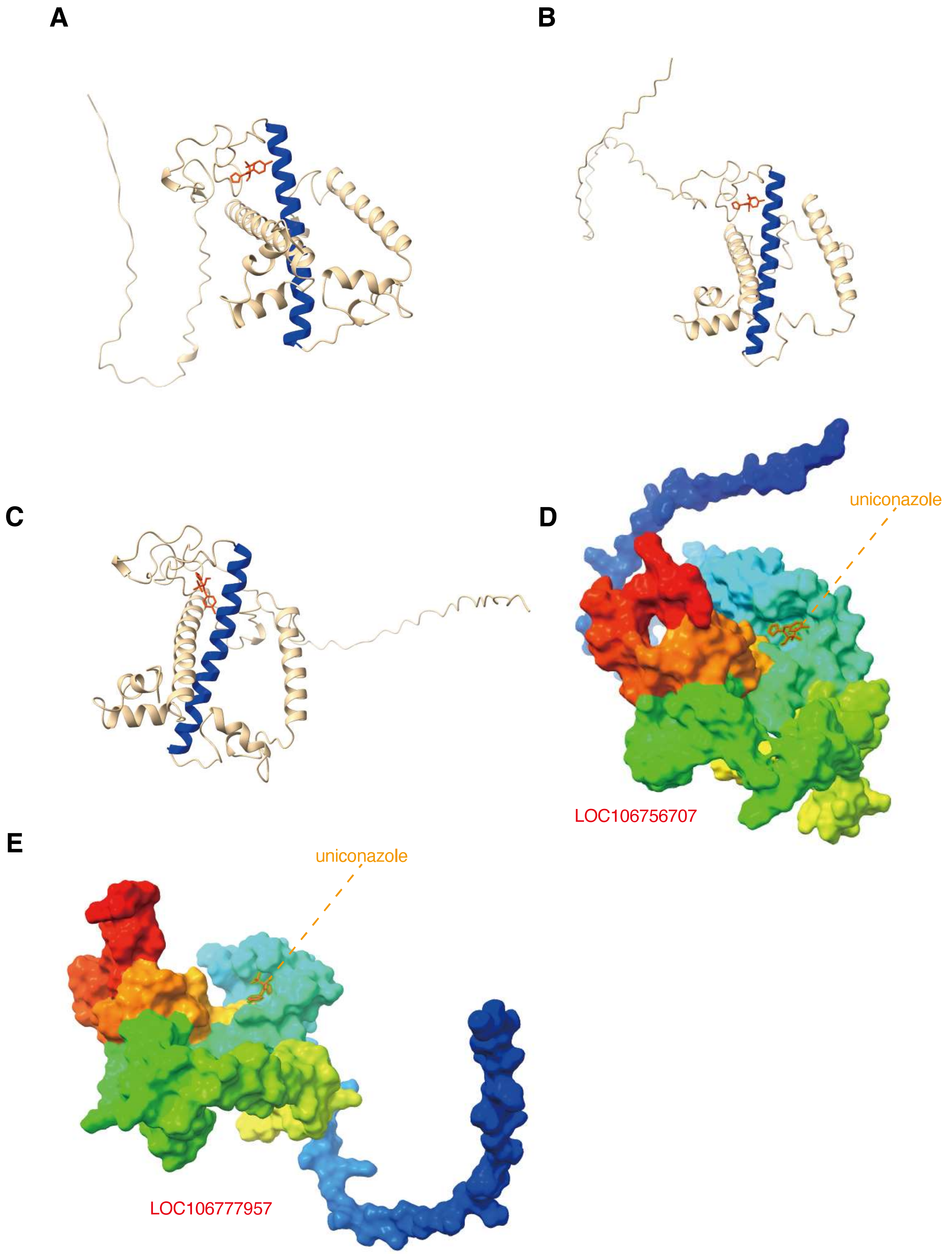

Fig.S6. The predicted relative postion of uniconazole and its potential targeting proteins. The neareast helix was highlighted as blue, and uniconazole was highlighted as orange.
