# Supplementary figures and images for: Comparative Analysis Highlights Uniconazole’s Efficacy in Enhancing the Cold Stress Tolerance of Mung Beans by Targeting Photosynthetic Pathways
Source: Plants (Basel). 2024 Jul 9;13(14):1885. doi: 10.3390/plants13141885 (PMC11280120; doi:10.3390/plants13141885)

A

starch content

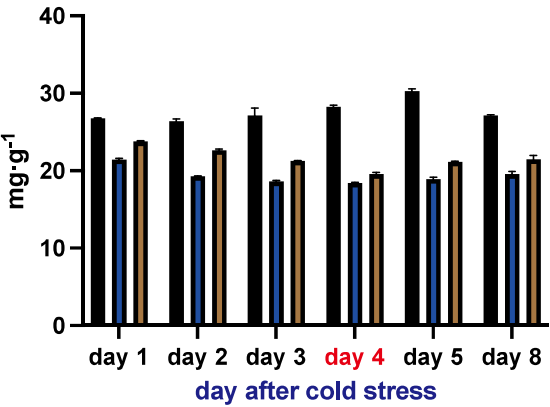

B

sucrose content

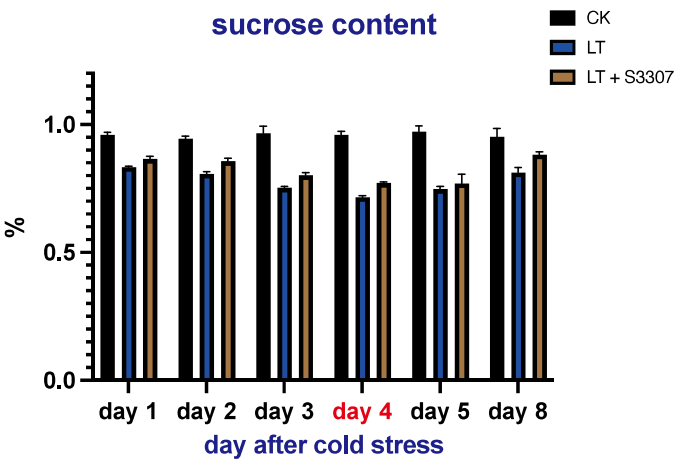

Supplement: Supplementary file 1 [file plants-13-01885-s001.zip › plants-3046194-supplementary-update/Figure-S1.pdf]

LT vs CK

## Soybean

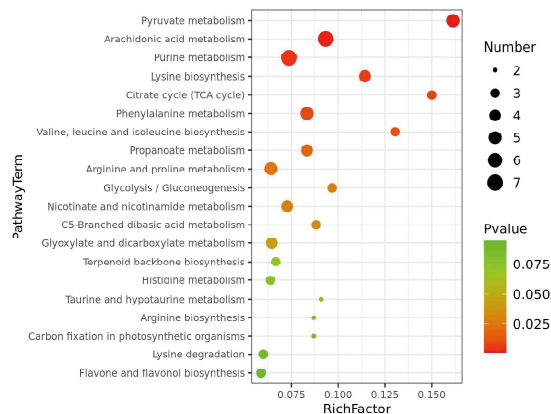

## Mungbean

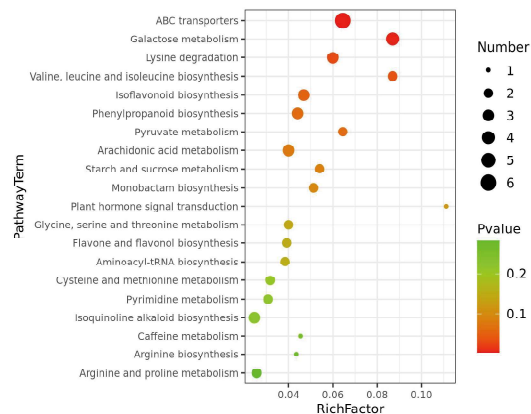

LT + S vs CK

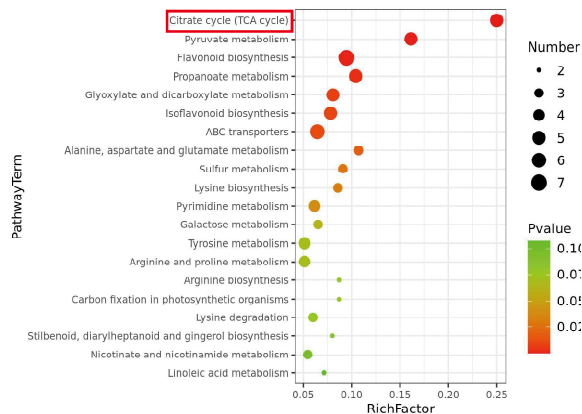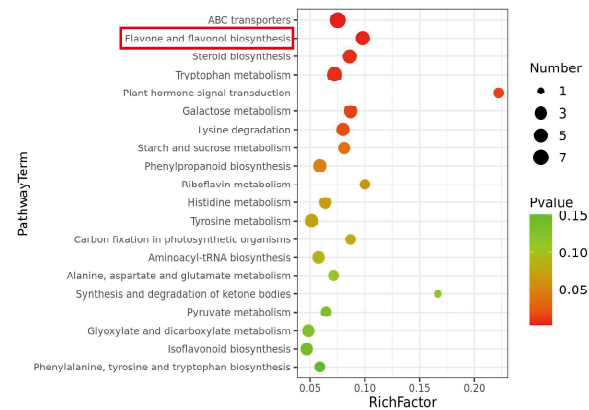

LT + S vs LT

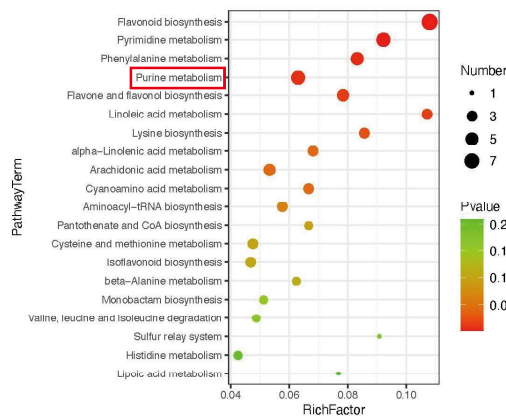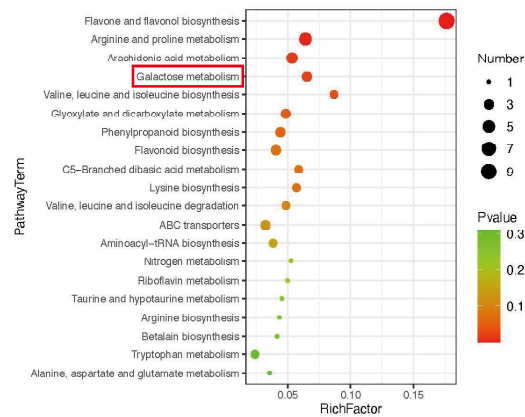

Supplement: Supplementary file 1 [file plants-13-01885-s001.zip › plants-3046194-supplementary-update/Figure-S2.pdf]

A

Top 20 up-regulated genes: mungbean

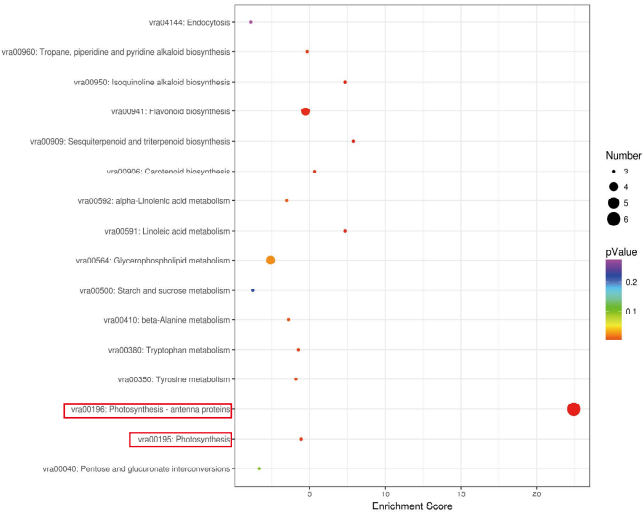

B

Top 20 down-regulated genes: soybean

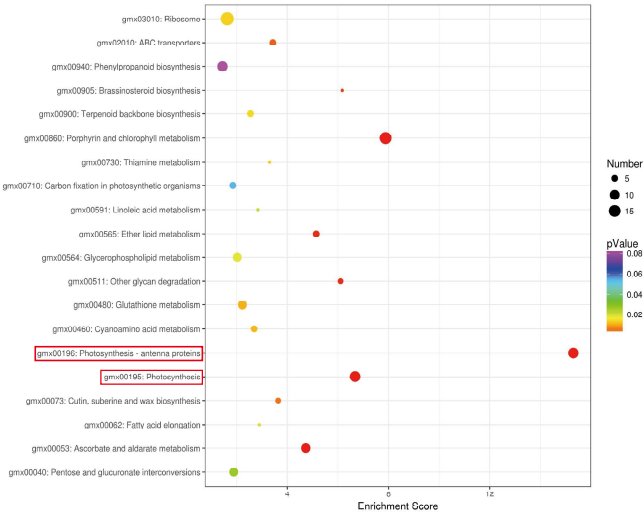

Supplement: Supplementary file 1 [file plants-13-01885-s001.zip › plants-3046194-supplementary-update/Figure-S3.pdf]

**A** Soybean

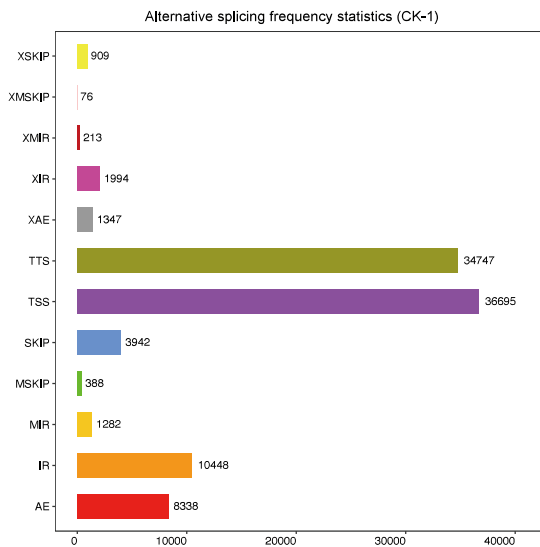

**B** Mung bean

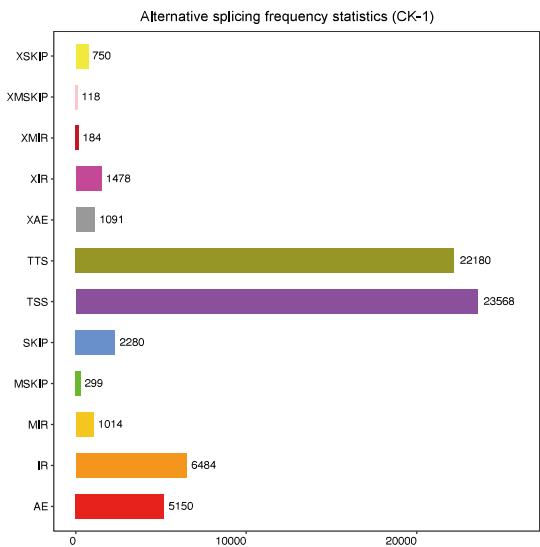

Supplement: Supplementary file 1 [file plants-13-01885-s001.zip › plants-3046194-supplementary-update/Figure-S4.pdf]

## Mungbean under cold stress

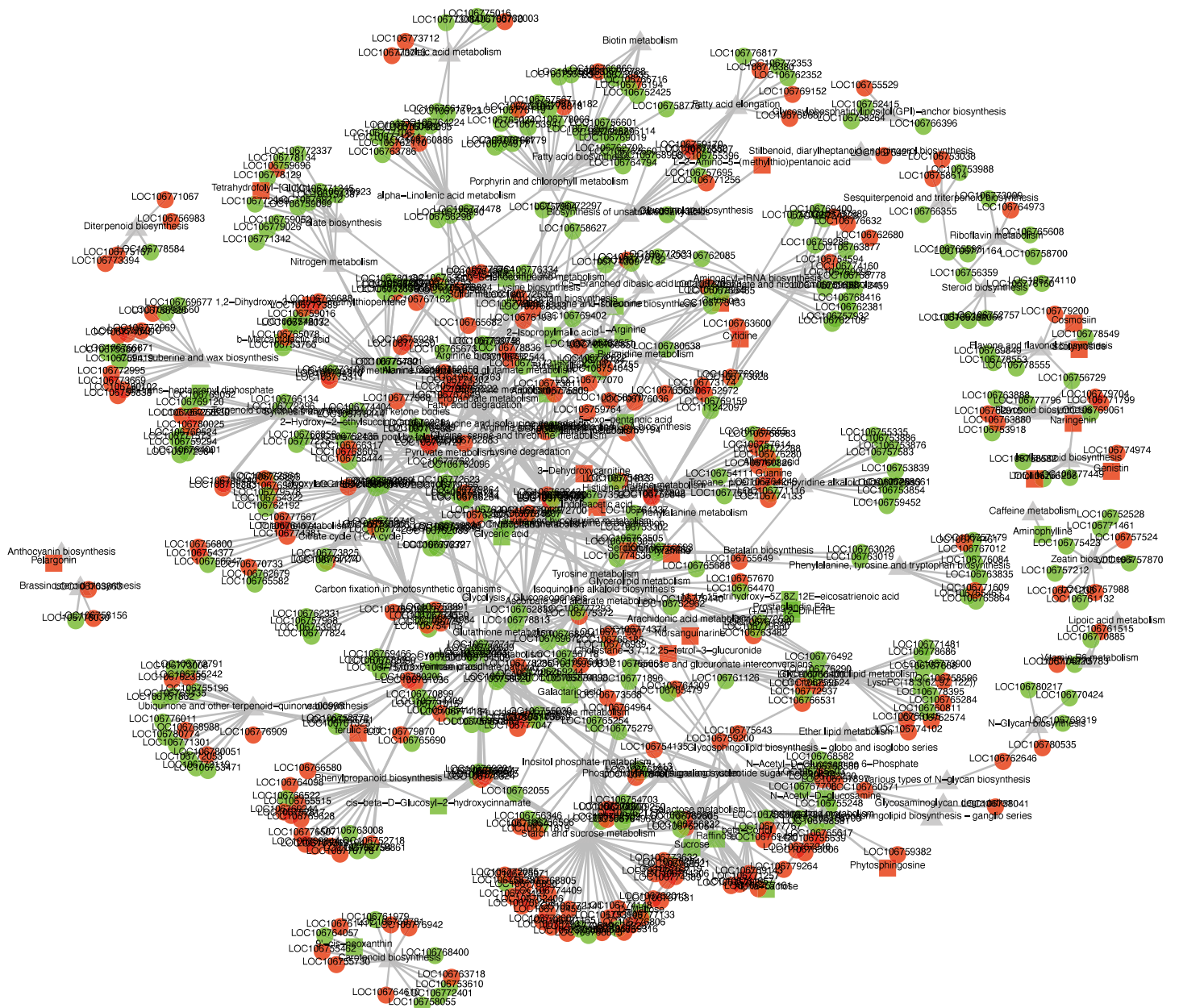

type ● gene ▲ map ■ Metabolites

Supplement: Supplementary file 1 [file plants-13-01885-s001.zip › plants-3046194-supplementary-update/Figure-S5.pdf]
